# Laccase-Catalyzed Derivatization of Aminoglycoside Antibiotics and Glucosamine

**DOI:** 10.3390/microorganisms10030626

**Published:** 2022-03-15

**Authors:** Annett Mikolasch, Ulrike Lindequist, Sabine Witt, Veronika Hahn

**Affiliations:** 1 Institute for Microbiology, University of Greifswald, Felix-Hausdorff-Str. 8, 17489 Greifswald, Germany; annett.mikolasch@uni-greifswald.de; 2 Interfaculty Institute for Genetics and Functional Genomics, University of Greifswald, Felix-Hausdorff-Str. 8, 17489 Greifswald, Germany; 3 Institute of Pharmacy, University of Greifswald, Friedrich-Ludwig-Jahn-Str. 17, 17487 Greifswald, Germany; lindequi@uni-greifswald.de; 4 Biometec, Walther-Rathenau-Str. 49a, 17489 Greifswald, Germany; witt@biometec.de; 5 Leibniz Institute for Plasma Science and Technology (INP), Felix-Hausdorff-Str. 2, 17489 Greifswald, Germany

**Keywords:** laccase, biotransformation, aminoglycoside antibiotics, cytotoxicity, antimicrobial activity, multidrug resistance (MDR), β-lactam antibiotics, methicillin-resistant *Staphylococcus aureus* (MRSA), antimicrobial resistance (AMR)

## Abstract

The increasing demand for new and effective antibiotics requires intelligent strategies to obtain a wide range of potential candidates. Laccase-catalyzed reactions have been successfully applied to synthesize new β-lactam antibiotics and other antibiotics. In this work, laccases from three different origins were used to produce new aminoglycoside antibiotics. Kanamycin, tobramycin and gentamicin were coupled with the laccase substrate 2,5-dihydroxy-*N*-(2-hydroxyethyl)-benzamide. The products were isolated, structurally characterized and tested in vitro for antibacterial activity against various strains of Staphylococci, including multidrug-resistant strains. The cytotoxicity of these products was tested using FL cells. The coupling products showed comparable and, in some cases, better antibacterial activity than the parent antibiotics in the agar diffusion assay, and they were not cytotoxic. The products protected mice against infection with *Staphylococcus aureus*, which was lethal to the control animals. The results underline the great potential of laccases in obtaining new biologically active compounds, in this case new antibiotic candidates from the class of aminoglycosides.

## 1. Introduction

Aminoglycoside antibiotics are oligomers closely related to carbohydrates. They are composed of a diaminocyclitol and amino sugars, or monosaccharides glycosidically linked to it. The amino groups of the components may be modified by methylation or amidination. Among the therapeutically important aminoglycoside antibiotics are the kanamycins, gentamicins and tobramycin important representatives which are produced by *Streptomyces* (kanamycins, tobramycin) and *Micromonospora* (gentamicins) species.

Aminoglycoside antibiotics bind to the 16S rRNA of the 30S subunit of ribosomes. This leads to the misreading of mRNA, and thus to the formation of incorrectly constructed proteins, resulting in cell death. They have a bactericidal effect on aerobic Gram-negative bacteria, including *Pseudomonas aeruginosa* and *Serratia marcescens*, and on the Gram-positive Staphylococci. Resistance is due to modification of the antibiotic by bacterial aminoglycoside-modifying enzymes (AME), which acylate, phosphorylate or adenylate the antibiotic. Resistance can also occur due to modification of the aminoglycoside-binding site of the ribosome [1].

Thanks to the combination of their high efficacy, low cost, lack of drug-related allergy, absence of interaction with other drugs and other advantages, aminoglycoside antibiotics are still among the most commonly used antibiotics worldwide. Aminoglycoside antibiotics are listed by the WHO as critically important antimicrobials for human therapy [2,3]. On the other hand, the development of resistance and cross-resistance, the relatively large nephro- and ototoxicity, and the need for parenteral application of aminoglycoside antibiotics to achieve a systemic effect require the development of novel compounds in which these disadvantages are avoided or reduced and efficacy is preserved or improved.

Several attempts have been made to achieve these goals, including the development of semisynthetic derivatives such as amikacin, the design of inhibitors of aminoglycoside-modifying enzymes [4], combinations with other antibiotics and the combination with adjuvants such as mannitol that reduce the effective dose of the antibiotic [5]. 

Laccase-mediated derivatization is a well-described method to synthesize novel antibiotics by enzymatic catalysis. The advantages of this approach are its low cost, mild reaction conditions and the high specificity of the laccase-initiated reactions [6]. The derivatization of β-lactam antibiotics has been of particular interest. Thus, Agematu et al. described the dimerization of various penicillin X esters [7] and the synthesis of cephalosporin antibiotics [8] by laccase-mediated reactions. In addition, in our former work, we have focused on the derivatization of approved β-lactam antibiotics [9,10,11,12,13], their core elements [14] and antibiotics with sulfonamide or sulfone structures [15]. Here, we describe, for the first time, the laccase-catalyzed derivatization of aminoglycoside antibiotics. In addition to the antibiotics kanamycin, tobramycin and gentamicin, the simpler structured glucosamine was used as a model compound.

## 2. Materials and Methods

### 2.1. Enzymes

Laccase was isolated from *Pycnoporus cinnabarinus* SBUG-M 1044 (PcL). This white rot fungus *P. cinnabarinus* was isolated from an oak tree in the North of Germany and deposited at the strain collection of the Institute for Microbiology of the Department of Biology of the University of Greifswald (SBUG), from which the strain was obtained. The cultivation of *P. cinnabarinus* SBUG-M 1044 and the preparation of its laccase were reported previously [16]. This enzyme preparation contains only isoenzymes of laccase, and no other enzymes. It was used in 20 mM sodium acetate buffer (SAB) pH 5.0, since the optimum pH is around pH 5.0 [16,17].

Laccase from *Myceliophthora thermophila* (MtL, expressed in genetically modified *Aspergillus* sp.) was obtained from Novozymes (Bagsvaerd, Denmark). It was used as received (activity 1000 U/g; substrate: syringaldazine) in citrate phosphate buffer (CPB, 18 mM citrate, 165 mM phosphate) at its optimum pH of pH 7.0 [18].

Extracellular laccase C of *Trametes spec.* (TsC) was obtained from ASA Spezialenzyme (Wolfenbüttel, Germany) and used in an activity of 800 nmol⋅ml^−1^⋅min^−1^ (substrate: 2,2′-amino-bis-3-ethylbenzthiazoline-6-sulfonic acid).

### 2.2. Measurement of Laccase Activity

The activity of laccase was measured spectrophotometrically at 420 nm with ABTS (2,2′-azino-bis(3-ethylbenzothiazoline-6-sulfonic acid) diammonium salt) as substrate [19] using the method described by Jonas et al. [16]. Here, 1 unit (U) of enzyme activity is defined as the amount of enzyme required for the oxidation of 1 µmol ABTS per min [19].

### 2.3. Experimental Procedures

Analytical procedure: For analytical experiments, glucosamine (**1a**) or one of the aminoglycosides (kanamycin (**1b**), tobramycin (**1c**) or gentamicin (**1d**; 1 mM, 2 mM, 5 mM, 10 mM)) and the laccase substrate 2,5-dihydroxy-*N*-(2-hydroxyethyl)-benzamide (**2a**; 1 mM, 2 mM) were incubated with each of the laccase preparations (activity 0.5 U) in separate assays. These reaction mixtures were shaken at 200 rpm on a bench shaker (IKA-Vibrax-VXR, Staufen, Germany) at room temperature (RT). The reaction mixtures were analyzed by analytical high-performance liquid chromatography (aHPLC). Chemicals were purchased from commercial suppliers: glucosamine hydrochloride, kanamycin sulfate, tobramycin and gentamicin sulfate from Sigma-Aldrich (Steinheim, Germany), and 2,5-dihydroxy-*N*-(2-hydroxyethyl)-benzamide from Midori Kagaku Co., (Tokyo, Japan).

The separation of the substances and products was achieved by RP18 column (endcapped, 5-µm, LiChroCART^®^ 125-4 RP18 column; Merck, Darmstadt, Germany) at a flow rate of 1 mL/min. A solvent system consisting of methanol (eluent A) and 0.1% phosphoric acid (eluent B), starting from an initial ratio of 10% A and 90% B and reaching 100% methanol within 18 min, was used.

### 2.4. Isolation Procedure of Product ***3a***

The glucosamine (**1a**; 5 mM) was dissolved in 60 mL CPB. After the addition of MtL (activity 0.5 U), 2,5-dihydroxy-*N*-(2-hydroxyethyl)-benzamide (**2a**) was added (6 ml of a 20 mM solution in CPB). The reaction mixture was incubated for 2 h at RT with agitation at 200 rpm.

Isolation steps were performed by solid-phase extraction with a RP18 silica gel column (C18-E (55 µm, 70 Å), 60 mL, 10 g adsorbent material, phenomenex, Strata, Aschaffenburg, Germany). After charging the column with 50 mL reaction mixture, the elution of the orange fraction was performed with 30 mL of a mixture of A. bidest. (double-distilled water) and methanol 1:1. The column was washed and regenerated with methanol. These steps were repeated until all of the reaction mixture was processed.

For mass spectrometry (MS) and nuclear magnetic resonance (NMR) spectroscopy, the isolated product **3a** was dried by lyophilization. 

### 2.5. Isolation Procedure of Products ***3b***, ***3c*** and ***3d***

First, 2 mM (240 mg) kanamycin sulfate (**1b**) was dissolved in 150 mL sodium acetate buffer pH5 (0.02 M) in a 500 mL round-bottom flask (transfer to the sonication bath for complete dissolution). To this reaction solution, 50 mg of laccase C was added and shaken until the laccase was completely dissolved. During this process, the reaction solution turned yellow. Then, 2 mM (78 mg) of 2,5-dihydroxy-*N*-(2-hydroxyethyl)-benzamide (**2a**) in 50 ml of sodium acetate buffer pH5 (0.02 M) was added to the reaction mixture to start the laccase catalyzed coupling reaction. The batch was stirred at 235 rpm and RT for 2.5 h.

In addition, 2 mM (374 mg) tobramycin (**1c**) and 1 mM (392 mg) gentamicin sulfate (**1d**) were dissolved, and laccase C was added in the same way as described above for **1b**. Then, 2 mM (78 mg) of **2a** in 50 mL of sodium acetate buffer, pH5 (0.02 M), was added to the reaction mixture of **1c**, and 1 mM (39 mg) of **2a** in 50 mL of sodium acetate buffer was added to the assay with **1d**. The batches were stirred at 235 rpm and RT for 1.5 h.

Isolation steps were also performed by solid-phase extraction with RP18 silica gel columns as described above. After charging the column with 50 mL reaction mixture, the elution of the orange fraction was performed with 30 mL of acetonitrile. The column was washed and regenerated with methanol. These steps were repeated until all reaction mixture was processed.

For MS and NMR spectroscopy, the isolated products were dried by lyophilization.

### 2.6. Lyophilization

For lyophilization, the diluted eluates of the solid-phase extraction (maximum methanol/acetonitrile content: 10%) were frozen overnight at −20 °C. After storage at −70 °C for at least 2 h, the samples were dried in the Alpha 1–4 freeze dryer (Christ, Osterode, Germany) under the following conditions:
Set point of the main drying    2 h at 5 °C, then 20 °CSet point freezing−20 °CSet point vacuum1.030 mbarSecurity printing2.560 mbar

Lyophilizates were transferred to vials and stored protected from light at 4 °C.

### 2.7. Analytical High-Performance Liquid Chromatography (aHPLC)

Samples of the incubation mixtures were analyzed by HPLC-UV/Vis detector for routine analyses [7].

### 2.8. Characterization of Biotransformation Products

Product **3a** was characterized by mass spectrometry (MS) using HPLC-MS (HPLC: Agilent 1100, Waldbronn, Germany; MS: Bruker Daltonics microTOF ESI-TOF-HRMS Massenspektrometer, Bremen, Germany). The separation of the substances and products was achieved by a Zorbax SB-C18 (2.1 × 50 mm, 1.8 μm) column (Agilent, Waldbronn, Germany) at a flow rate of 0.5 mL/min. A solvent system consisting of acetonitrile (eluent A) and 0.1% formic acid (eluent B), starting from an initial ratio of 10% A and 90% B and reaching 100% acetonitrile within 14 min, was used. 

Products **3b**, **3c** and **3d** were characterized by MS using electrospray ionization under atmospheric conditions (API-ES) (drying and nebulizing gas: nitrogen) on a Bruker-Daltonics microTOF instrument (Bremen, Germany).

The NMR spectra were recorded on a Bruker Avance 600 instrument (Rheinstetten, Germany) at 600 MHz. The solvents used were H_2_O-d2 and DMSO-d6. Chemical shifts are expressed in δ (ppm) calibrated on the resonances of the residual non-deuterated solvent. J values are given in Hz. 

### 2.9. Characterization of Glucosamine ***1a*** by NMR

Since the glucosamine used was a mixture of α-D-glucosamine and β-D-glucosamine, it was characterized by NMR. For the following structural determinations, these data were compared to the NMR data described by Breitmaier and Voelter [20]. 

α-D-glucosamine: ^1^H (D_2_O) δ (ppm) 3.31 (dd, ^3^J_ae_ = 3.7 Hz, 1H, H-2, α-D-g), 3.50 (m, ^3^J = 9.5 Hz, 1H, H-3, α-D-g), 3.79 (m, ^3^J = 5.2 Hz, ^3^J = 12.3 Hz, 2H, H-6, α-D-g), 3.90 (broad, H-5, α-D-g), 5.45 (d, ^3^J_ae_ = 3.7 Hz, 1H, H-1, α-D-g); ^13^C (D_2_O) δ (ppm) 57.15 (C-2, α-D-g), 63.22 (C-6, α-D-g), 72.42 (C-3, α-D-g), 72.45 (C-4, α-D-g), 74.43 (C-5, α-D-g), 91.93 (C-1, α-D-g); HMBC H-1 (72.42 (C-3, α-D-g), 74.43 (C-5, α-D-g)), H-2 (72.42 (C-3, α-D-g), 91.93 (C-1, α-D-g)), H-3 (57.15 (C-2, α-D-g), 72.45 (C-4, α-D-g)), H-6 (74.43 (C-5, α-D-g)). 

β-D-glucosamine: ^1^H (D_2_O) δ (ppm) 3.02 (dd, ^3^J_aa_ = 8.5 Hz, ^3^J = 10.6 Hz, 1H, H-2, β-D-g), 3.53 (m, ^3^J = 2.3 Hz, ^3^J = 5.6 Hz, 1H, H-5, β-D-g), 3.70 (dd, ^3^J = 8.6 Hz, ^3^J = 10.6 Hz, 1H, H-3, β-D-g), 3.75 (m, ^3^J = 5.5 Hz, ^3^J = 12.4 Hz, 2H, H-6, β-D-g), 4.95 (d, ^3^J_aa_ = 8.5 Hz, 1H, H-1 β-D-g); ^13^C (D_2_O) δ (ppm) 59.61 (C-2, β-D-g), 63.35 (C-6, β-D-g), 72.56 (C-4, β-D-g), 74.84 (C-3, β-D-g), 78.97 (C-5, β-D-g), 95.54 (C-1, β-D-g); HMBC H-1 (59.61 (C-2, β-D-g), 72.56 (C-4, β-D-g), 74.84 (C-3, β-D-g), 78.97 (C-5, β-D-g)), H-2 (74.84 (C-3, β-D-g), 95.54 (C-1, β-D-g)), H-3 (59.61 (C-2, β-D-g), 72.56 (C-4, β-D-g)), H-5 (72.56 (C-4, β-D-g), 74.84 (C-3, β-D-g), H-6 (78.97 (C-5, β-D-g)). 

### 2.10. Determination of Antibacterial Activity 

An agar diffusion method, previously described by Mikolasch et al. [10], was used to determine the antibacterial activity in the range from 0.0127 to 0.127 µmol. The bacterial strains *Staphylococcus aureus* (*S. aureus*) ATCC 6538/DSM 799 and the multi-resistant strains isolated from patients *S. aureus* 34289, *S. aureus* 36881, *S. aureus* 38418, *Staphylococcus epidermidis* (*S. epi.*) 125, *S. epidermidis* 847 and *Staphylococcus haemolyticus* (*S. haem.*) 535 were used.

### 2.11. Cytotoxic Activity

Cytotoxicity was determined by a neutral red uptake assay using FL cells, a human amniotic epithelial cell line, as reported previously [10]. 

### 2.12. Animal Assays

A “*Staphylococcus*-infected, immune suppressed mouse” model, as described by Mikolasch et al. [10], was used for the examination of in vivo effectiveness of in vitro active products.

## 3. Results

### 3.1. Analytical Screening of Aminoglycosides, Glucosamine and Laccases

Glucosamine (**1a**) and the different aminoglycosides kanamycin (**1b**), tobramycin (**1c**), gentamicin (**1d**) (1 mM, 2 mM, 5 mM and 10 mM) and the laccase substrate 2,5-dihydroxy-*N*-(2-hydroxyethyl)-benzamide (**2a**; 1 mM, 2 mM) were subjected to laccase-catalyzed transformations with three kinds of laccase (Table 1).

The simpler structured **1a** was composed of a sugar ring and a single amino group. Since the glucosamine used was a mixture of α-D-glucosamine and β-D-glucosamine, as indicated by the NMR data, it was expected that at least two heteromolecular dimers could be formed. For all assays performed with **1a**, more than two heteromolecular products were detected by aHPLC, regardless of the concentration of the educts **1a** and **2a** and the type of laccase. However, the reaction involving 5 mM **1a** and 1 mM laccase substrate **2a** produced only one main product (Table 1), making the structural elucidation of the product straightforward. After isolation by solid-phase extraction, the structural elucidation was performed using MS and NMR data (see Section 3.2).

The more complex structures of **1b**, **1c** and **1d** consisted of three sugar rings and at least three amino groups. Furthermore, **1a** and **1c** consisted of only one structural molecule, whereas **1b** and **1d** were mixtures of kanamycin A, kanamycin B and kanamycin C, and gentamicin C_1_, gentamicin C_2_, gentamicin C_1a_, gentamicin C_2a_ and gentamicin C_2b_. With the mixtures of **1b** and **1d**, there were multiple ways of coupling the laccase substrate to the aminoglycoside, resulting in a variety of products. Indeed, in these cases, a mixture of more than 10 products was detected by aHPLC independent of the source of laccase. The grey-shaded examples in Table 1 were selected for product isolation. After isolation by solid-phase extraction, the structural elucidation was performed by MS.

### 3.2. Structural Elucidations

**Figure 1:**
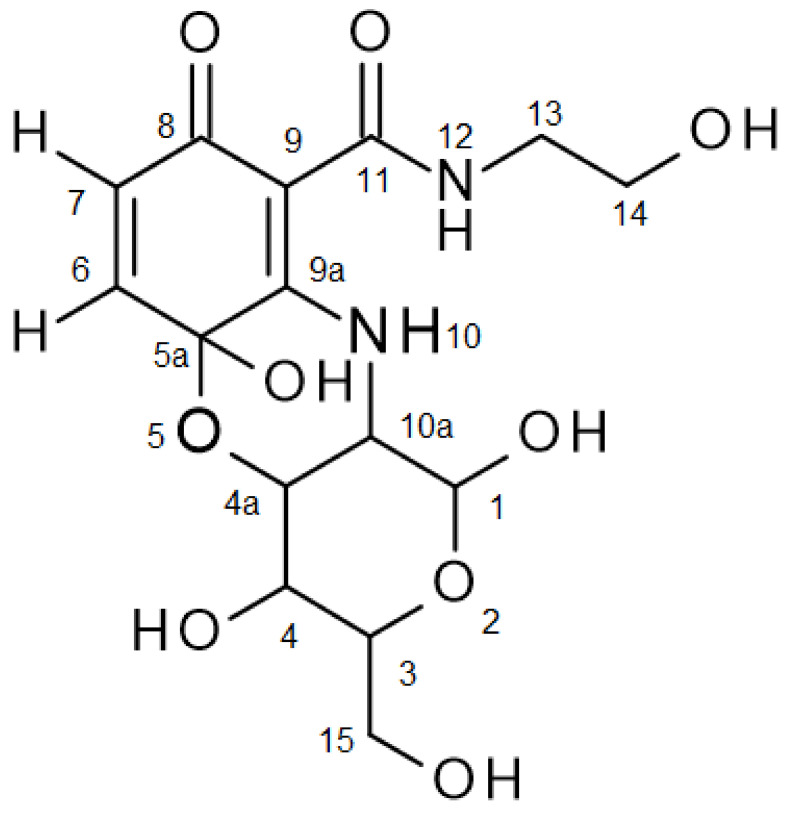
Product numbering of **3a**.

Product **3a** showed two absorption maxima under 270 nm (235 nm, 259 nm), one at 343 nm and a minor one at 470 nm. The MS measurement in positive mode showed a molecular mass of m/z 373.124936 for **3a**, calculated with 372.116880 (M^+^) error −2.11 ppm, corresponding to the product structure of Figure 1. This mass can be attributed to a coupling of the amino group of **1a** on the quinonoid form of **2a** and a cyclization via a hydroxyl group of **1a** on the carbonyl group (C-5a) of the quinone, resulting in a six-membered non-aromatic ring (Figure 1). ^1^H NMR spectral data of **3a** showed characteristic signals for both **1a** and **2a** (Figure 1 and Table 2). The multiplicity of H-6 and H-7 suggests that the first step of dimerization occurred at C-9a. The chemical shift of the proton at N-10 was shifted to the lower field. The HMBC correlations to C-5a, C-9, C-9a and C-10a (Figure 1 and Table 2) of the proton H-10 unambiguously fixed the amination at C-9a. A broad signal for the phenolic hydroxyl group at C-5a at around 7.5 ppm, and the HMBC correlation of H-4a to C-5a (86.73 ppm), support the concept of cyclization of the C-4a hydroxyl group at C-5a and the removal of the *para*-quinonoid character of **3a**. In addition, ^13^C NMR showed only one typical signal of a quinonoid structure in the region of 180 ppm, indicating only one carbonyl group at C-8. The HMBC spectrum also showed correlations between the proton H-7 and C-5a, and between the proton H-6 and the carbonyl carbon C-8 and the C-9a, unambiguously showing **3a** to be the aminated and cyclized product 1,4,5a-trihydroxy-*N*-(2-hydroxyethyl)-3-(hydroxymethyl)-8-oxo-1,3,4,4a,10,10a-hexahydropyrano [4,3-b][1,4]benzoxazine-9-carboxamide. Since the glucosamine used contained the forms α-D-glucosamine and β-D-glucosamine, the product **3a** was also a mixture of **3a_1_** α-D-glucosamine product and **3a_2_** β-D-glucosamine product, which was clearly demonstrated by the NMR data of the substrate **1a** (see Section 2.9) and the NMR data of **3a** (Figure 1 and Table 2).

Product mixtures of **3b**, **3c** and **3d** were measured by MS analyses. Whereas **1b** and **1d** were mixtures of kanamycin A, kanamycin B and kanamycin C, and gentamicin C_1_, gentamicin C_2_, gentamicin C_1A_, gentamicin C_2a_ and gentamicin C_2b_, **1c** consisted of only one substance, which resulted in fewer product possibilities, and hence a simpler product analysis for **3c**. The MS measurement of **3c** with AP-ESI in both positive and negative modes showed the molecular mass of **3c** to be 660 (MS m/z AP-ESI: neg. mode [M-H]^−^ 659.2965; AP-ESI: pos. mode [M+H]^+^ 661.3415). These MS data lead to the conclusion that heteromolecular dimers were formed comparable to the structure of **3a**. 

MS measurements on **3b** and **3d** did not result in clear structural assignments, since a wide range of hypothetical products were possible due to the different amino and hydroxyl groups, and thus to multiple coupling options. For example, the gentamicin preparation consisted of the components C_1_ (3 free amino groups), C_2_ (4 free amino groups), C_1A_ (4 free amino groups), C_2a_ (4 free amino groups) and C_2b_ (3 free amino groups), so 18 amino groups were available for the formation of a covalent bond to the carbon atoms of laccase substrate **2a**. Because of this, there was a possibility for the formation of various dimers and higher molecular weight structures, such as tri- and tetramers, as previously described [16,21,22,23,24,25,26,27,28]. However, some statements about possible structures could be drawn from the NMR studies. For both **3b** and **3d**, the signals for H-6, H-7, H-10 and H-12 corresponding to that of structure **3a** could be analyzed. Moreover, many CH signals of the glycosidic structural part were detected by ^1^H NMR spectra (Table 3), indicating coupling products comparable to **3a**. 

### 3.3. Biological Activity of the Biotransformation Products

The product mixtures from **3b** to **3d** showed all a moderate to strong growth inhibition of several Gram-positive strains, including multidrug-resistant *Staphylococcus* strains, in the agar diffusion assay (Table 4). 

Analyses of the stability of the products showed a limited lifetime in aqueous solution. Incubation of the solutions with **3a** to **3d** at 30 °C showed decomposition after 4 h. Therefore, the survey of the antimicrobial effects was concentrated on the initial screening using the agar diffusion test. 

In addition to the antimicrobial efficacy, the cytotoxicity against FL cells was tested. The results showed that **3a**–**3d**, as well as **1a**–**1d** and **2a**, showed no cytotoxic effect at concentrations of 12.5, 25, 50 and 100 µg/mL. The growth of FL cells was comparable with the control culture (100–96% vitality).

A “Staphylococcus-infected, immune suppressed mouse” model was used for the examination of in vivo effectiveness of in vitro active products (Table 5). With one exception, all mice treated i.p. with one of the biotransformation product mixtures from **3b** to **3d** survived infection with *Staphylococcus aureus* ATCC 6538, whereas all untreated mice died after infection within 2 d. The treated and surviving mice did not show any signs of intoxication. 

## 4. Discussion

### 4.1. Biotransformation of Glucosamine and Aminoglycosides

Reaction mechanism of glucosamine (**1a**) with 2,5-dihydroxy-*N*-(2-hydroxyethyl)-benzamide (**2a**). 

**Figure 2:**
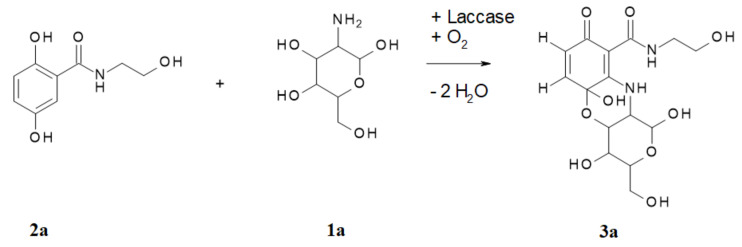
Laccase-catalyzed reaction of 2,5-dihydroxy-*N*-(2-hydroxyethyl)-benzamide (**2a**) with glucosamine (**1a**) for the synthesis of the product **3a**.

In the laccase-catalyzed reaction of **2a** with **1a**, the cyclisation product 1,4,5a-trihydroxy-*N*-(2-hydroxyethyl)-3-(hydroxymethyl)-8-oxo-1,3,4,4a,10,10a-hexahydropyrano[4,3-b][1,4]benzoxazine-9-carboxamide (**3a**) was readily produced by two laccase-mediated bond formations between **2a** and **1a** (Figure 2).

**Figure 3:**
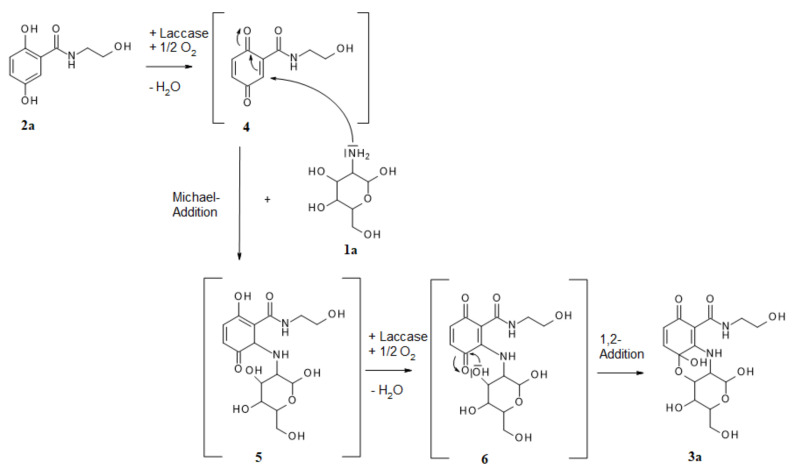
Possible reaction mechanism of laccase-mediated reaction for the synthesis of product **3a**.

The synthesis of the heteromolecular cyclic product **3a** can be described as a regioselective domino reaction. The first step was the laccase-mediated oxidation of **2a**, with oxygen resulting in a quinonoid transformation intermediate **4**, which then underwent an amination by intermolecular Michael-addition (1,4-addition; Figure 3).

The amination was affected by the amino group of **1a**. The position of this first amination step was the *ortho*-position to the carboxyl group of **2a**, as described for other aminations with very differently structured amines [6,9,10,21,29,30], resulting in intermediate **5**. After a second laccase-mediated oxidation, the unstable mono-aminated intermediate **6** was formed, which underwent an intramolecular 1,2-addition whereby the oxygen atom of the hydroxyl group 1 of **1a** formed an O-C bond at one quinonoid carbonyl group, resulting in **3a**, as described previously for the oxygen atom of 2-aminophenol, as well as sulphur [31] or nitrogen atoms [24]. However, the laccase-mediated cyclisation of 2,5-dihydroxybenzoic acid derivatives with glucosamines via N-C and O-C bond formations is here described for the first time.

The complex structures of kanamycin **1b**, tobramycin **1c** and gentamicin **1d** consisted of three sugar rings and at least three amino groups, so that different functional amino and hydroxyl groups could react with substrate **2a**. In addition, a wide variety of products was formed, as described above (Table 1). However, since all groups were amino and hydroxyl groups on sugar rings, all resulting products had comparable structures to product **3a**. However, their structures were far more complex due to the presence of multiple sugar rings and functional groups.

### 4.2. Biological Activity of the Biotransformation Products

All products obtained by the coupling of an aminoglycoside antibiotic **1b** to **1d** with laccase substrate **2a** showed moderate to strong growth inhibition of several Gram-positive bacterial strains, including multidrug-resistant strains. As expected, the products prepared from the model compound glucosamine were not active.

In most cases, the efficacy was in the same range as that of the parent antibiotics. However, it is striking that product **3d**, derived from gentamicin, was also effective against strains that were resistant to gentamicin (*Staphylococcus epidermidis* 847, 125). This product also showed effects against the broadest spectrum of test strains. Product **3c**, obtained starting from tobramycin, showed slightly higher efficacy against *Staphylococcus epidermidis* 125 than tobramycin itself.

It is noteworthy that all coupling products resulting from the antibiotics, as well as the parent antibiotics themselves, were also active in vivo and, with one exception, were capable of protecting all infected mice. In contrast, all non-treated animals died from the infection. A very positive aspect is also the lack of cytotoxicity of the compounds.

In order to prove the potential benefits of the newly synthesized compounds, it will be necessary to include Gram-negative bacteria as test strains, to determine the minimum inhibitory concentrations, in addition to the agar diffusion test, to increase the number of test animals for the in vivo experiments and expand the toxicity tests.

In conclusion, the general suitability of laccases to catalyze the derivatization of aminoglycoside antibiotics was demonstrated. The derivatization processes occurred under mild reaction conditions using aqueous solvent systems, atmospheric pressure and room temperature. The obtained products possessed antimicrobial activity.

With this research, the potential of laccases for the synthesis of new antibiotic substances from various structural groups–β-lactam antibiotics [6,7,8,9,10,13], sulfonamide antibiotics [15], corollosporins [11], morpholines [32], mitomycins [33], phenoxazinones [34,35,36], catecholthioether derivatives [37], naphthoquinone sulfides [38] and now also aminoglycosides has been confirmed and extended. The use of laccases thus provides a suitable and cost-effective way to expand the spectrum of new and effective antibiotic candidates.

## Figures and Tables

**Table 1:** Glucosamine and aminoglycosides (**1a**–**1d**) used in laccase-catalyzed reactions as coupling partners, 2,5-dihydroxy-*N*-(2-hydroxyethyl)-benzamide (**2a**) used as laccase substrate and the number of synthesized heteromolecular products.

Coupling Partner		Laccase Substrate 2a	MtL	PcL	TsC
	Concentration	Concentration	Number of Heteromolecular Products		
**1a** Glucosamine	1 mM	1 mM	6	5 (+MP) ^(a)^	5 (+MP)
	2 mM	2 mM	6	Nep ^(b)^	nep
	5 mM	1 mM	1 (+MP)	7	7
	10 mM	1 mM	nep	6 (+MP)	nep
**1b** Kanamycin	1 mM	1 mM	>10	>10	>10
	2 mM	2 mM	>10	nep	>10
	5 mM	1 mM	>10	>10	>10
	10 mM	1 mM	nep	>10	nep
**1c** Tobramycin	1 mM	1 mM	>10	>10	>10
	2 mM	2 mM	>10	nep	>10
	5 mM	1 mM	>10	>10	>10
	10 mM	1 mM	nep	>10	nep
**1d** Gentamicin	1 mM	1 mM	>10	>10	>10
	2 mM	2 mM	>10	nep	nep
	5 mM	1 mM	>10	>10	>10
	10 mM	1 mM	nep	>10	nep

^(a)^ (+MP) = And minor products. ^(b)^ nep = No experiment performed. Grey-shaded = Approach used for product isolation.

**Table 2:** ^1^H assignments, and HSQC and HMBC correlations for **3a**.

^1^H Assignments	HSQC Correlations	HMBC Correlations
**3a_1_** α-D-glucosamine product		
3.27 ^(a)^ (m, ^3^J *=* 5.6 Hz, 4H, H-13)	40.67 ^(a)^ (C-13)	59.73 ^(a)^ (C-14), 168.85 (C-11)
3.43 (m, 1H, H-4)	68.77 (C-4)	70.83 (C-4a)
3.45 (t, ^3^J *=* 5.6 Hz, 4H, H-14)	59.73 (C-14)	40.67 (C-13)
3.73 (m, 1H, H-3)	73.63 (C-3)	52.33 (C-10a), 68.77 (C-4), 88.94 (C-1)
4.05 (m, J = 2.5 Hz, J = 10.8 Hz, 1H, H-10a)	52.33 (C-10a)	73.63 (C-3)
5.45 (d, ^3^J = 3.2 Hz, 1H, H-1)	88.94 (C-1)	73.63 (C-3)
5.85 (d, ^3^J *=* 10.1 Hz, 1H, H-7)	126.15 (C-7)	86.73 (C5a), 94.95 (C-9), (168.85 (C-11)) ^(^^b)^, ((182.21 (C-8))) ^(^^c)^
6.50 (d, ^3^J *=* 10.1 Hz, 1H, H-6)	138.64 (C-6)	((86.73 (C-5a))), ((94.95 (C-9))), 165.90 C-9a, 182.21 (C-8)
9.75 (s(broad), 2H, H-12)	-	40.67 (C-13), 59.73 (C-14), (94.95 (C-9), 168.85 (C-11)
11.84 (s, 1H, NH, H-10)	-	52.33 (C-10a), 86.73 (C-5a), 88.94 (C-1), 94.95 (C-9), ((165.90 C-9a))
**3a_2_** β-D-glucosamine product		
3.27 (m, ^3^J *=* 5.6 Hz, 2H, H-13)	40.67 (C-13)	59.73 (C-14), 168.85 (C-11)
3.43 (m, 1H, H-4)	68.77 (C-4)	70.83 (C-4a)
3.45 (t, ^3^J *=* 5.6 Hz, 2H, H-14)	59.73 (C-14)	40.67 (C-13)
3.52 (m, 2H, H-4a, H-3)	70.83 (C-4a), 76.68 (C-3)	54.64 (C-10a), 68.77 (C-4), 76.68 (C-3), 86.73 (C-5a), 93.66 (C-1)
3.62 (d, J *=* 10.3 Hz, 2H, H-15)	63.37 (C-15)	68.77 (C-4), 76.68 (C-3)
3.74 (m, 1H, H-10a)	54.64 (C-10a)	93.66 (C-1)
4.66 (d, ^3^J = 7.9 Hz, 1H, H-1)	93.66 (C-1)	54.64 C-10a, 76.68 C-3)
5.87 (d, ^3^J *=* 10.1 Hz, 1H, H-7)	126.15 (C-7)	86.73 (C5a), 94.95 (C-9), (168.85 (C-11)), ((182.21 (C-8)))
6.51 (d, ^3^J *=* 10.1 Hz, 1H, H-6)	138.64 (C-6)	((86.73 (C-5a))), ((94.95 (C-9))), 165.90 C-9a, 182.21 (C-8)
9.75 (s(broad), 2H, H-12)	-	40.67 (C-13), 59.73 (C-14), (94.95 (C-9), 168.85 (C-11)
11.90 (s, 1H, NH, H-10)	-	54.64 (C-10a), (68.77 (C-4)), (76.68 (C-3)), 86.73 (C-5a), 94.95 (C-9), (165.90 C-9a)

^(a)^ Chemical shifts are expressed in d(ppm) calibrated on the resonances of the residual non-deuterated solvent DMSO. J values are in Hz. ^(b)^ Signals with low intensity. ^(c)^ Signals with very low intensity.

**Table 3:** ^1^H assignments for **3b** and **3d** in comparison to **3a_1_**.

3a_1_ α-D-Glucosamine Product	3b Kanamycin Product	3d Gentamicin Product
3.27–5.45 ppm CHs of the glycosidic structural part	3–6 ppm CHs of the glycosidic structural part	3–6 ppm CHs of the glycosidic structural part
5.85 ^(a)^ (d, ^3^J *=* 10.1 Hz, 1H, H-7)	6.38 (d, ^3^J *=* 10.2 Hz, 1H, H-7)	6.52 (d, ^3^J *=* 10.2 Hz, 1H, H-7)
6.50 (d, ^3^J *=* 10.1 Hz, 1H, H-6)	6.57 (d, ^3^J *=* 10.2 Hz, 1H, H-6)	6.67 (d, ^3^J *=* 10.2 Hz, 1H, H-6)
9.75 (s(broad), 2H, H-12)	9.73 (s(broad), 2H, H-12)	9.72 (s(broad), 2H, H-12)
11.84 (s, 1H, NH, H-10)	12.98 (s, 1H, NH, H-10)	13.16 (s, 1H, NH, H-10)

^(a)^ Chemical shifts are expressed in d (ppm) calibrated on the resonances of the residual non-deuterated solvent DMSO. J values are in Hz.

**Table 4:** Antimicrobial activity of products **3a**–**3d**, and educts **2a** and **1a**–**1d**.

Compound	Amount [µmol]	*S. aureus* ATCC 6538	*S. aureus* 34289 ^(d)^	*S. aureus* 36881 ^(d)^	*S. aureus* 38418 ^(d)^	*S. aureus* 315 ^(d)^	*S. epi.* 847 ^(d)^	*S. epi.* 125 ^(d)^	*S. haem.* 535 ^(d)^
**1a**	0.127	r ^(a)^	r	r	r	r	r	r	r
	0.063	r	r	r	r	r	r	r	r
	0.0127	r	r	r	r	r	r	r	r
**1b**	0.127	24 ^(b)^	r	r	24	r	r	r	16
		(1.5) ^(c)^			(1.6)				(0.6)
	0.063	20	r	r	20	r	r	r	r
		(0.6)			(0.8)				
	0.0127	16	r	r	12	r	r	r	r
		(1.0)			(1.0)				
**1c**	0.127	24	r	26	28	r	r	12	22
		(0.6)		(1.2)	(1.0)			(1.6)	(0.6)
	0.063	22	r	24	24	r	r	10	20
		(0.4)		(0.2)	(0.8)			(0.6)	(0.9)
	0.0127	16	r	16	18	r	r	r	r
		(1.0)		(1.4)	(1.0)				
**1d**	0.127	24	26	26	26	26	r	r	18
		(1.1)	(0.9)	(1.8)	(1.6)	(1.1)			(0.6)
	0.063	22	24	22	22	24	r	r	16
		(0.8)	(0.8)	(1.3)	(1.2)	(1.8)			(1.5)
	0.0127	16	22	20	20	20	r	r	10
		(1.3)	(1.1)	(0.8)	(0.6)	(1.0)			(0.6)
**2a**	0.127	r	r	r	r	r	r	r	r
	0.063	r	r	r	r	r	r	r	r
	0.0127	r	r	r	r	r	r	r	r
**3a**	0.127	r	r	r	r	r	r	r	r
	0.063	r	r	r	r	r	r	r	r
	0.0127	r	r	r	r	r	r	r	r
**3b**	0.127	24	r	r	22	r	r	r	10(0.0)
		(1.3)			(0.6)				r
	0.063	20	r	r	20	r	r	r	
		(0.9)			(0.7)				r
	0.0127	14	r	r	14	r	r	r	
		(1.0)			(1.4)				
**3c**	0.127	26	r	26	26	r	r	20	22
		(1.0)		(1.2)	(1.0)			(1.6)	(0.4)
	0.063	24	r	24	24	r	r	16	20
		(0.4)		(0.2)	(0.8)	r		(0.8)	(1.4)
	0.0127	20	r	18	20		r	r	r
		(0.6)		(1.0)	(1.4)				
**3d**	0.127	24	28	26	26	28	10	12	18
		(1.6)	(1.0)	(2.0)	(1.6)	(1.2)	(1.7)	(1.5)	(0.6)
	0.063	22	24	22	22	24	r	r	16
		(0.6)	(0.8)	(1.5)	(1.2)	(1.8)			(1.5)
	0.0127	18	20	20	20	18	r	r	10
		(1.2)	(1.0)	(0.6)	(0.6)	(1.0)			(0.6)

^(a)^ Resistant (no zone of inhibition). ^(b)^ Zones of inhibition (mm) calculated from 3 replicates. ^(c)^ Standard deviation calculated from 3 replicates. ^(d)^ Multidrug-resistant strains.

**Table 5:** Effectiveness of in vitro active products in the “*Staphylococcus*-infected, immune suppressed mouse” model–*Staphylococcus aureus* ATCC 6538.

Compound	Dose	Survived/Treated Mice n/n	Survived/Control Mice n/n
**1b**	2 × 1.0 mg (50 mg/kg)	3/3	0/5
**1c**	2 × 1.0 mg (50 mg/kg)	3/3	0/5
**1d**	2 × 1.0 mg (50 mg/kg)	3/3	0/5
**3b**	2 × 1.0 mg (50 mg/kg)	2/3	0/5
**3c**	2 × 1.0 mg (50 mg/kg)	3/3	0/5
**3d**	2 × 1.0 mg (50 mg/kg)	3/3	0/5
